# Machine learning based DNA melt curve profiling enables automated novel genotype detection

**DOI:** 10.1186/s12859-024-05747-0

**Published:** 2024-05-10

**Authors:** Aaron Boussina, Lennart Langouche, Augustine C. Obirieze, Mridu Sinha, Hannah Mack, William Leineweber, April Aralar, David T. Pride, Todd P. Coleman, Stephanie I. Fraley

**Affiliations:** 1 Division of Biomedical Informatics, University of California San Diego, La Jolla, CA 92093 USA; 2 Department of Nanoengineering, University of California San Diego, La Jolla, CA 92093 USA; 3 Department of Bioengineering, University of California San Diego, La Jolla, CA 92093 USA; 4 Department of Pathology, University of California San Diego, La Jolla, CA 92093 USA; 5 Department of Bioengineering, Stanford University, Stanford, CA 94305 USA

**Keywords:** Melt curve, Machine learning, Pathogen identification, 16S rRNA, Novelty detection, Emerging pathogens

## Abstract

Surveillance for genetic variation of microbial pathogens, both within and among species, plays an important role in informing research, diagnostic, prevention, and treatment activities for disease control. However, large-scale systematic screening for novel genotypes remains challenging in part due to technological limitations. Towards addressing this challenge, we present an advancement in universal microbial high resolution melting (HRM) analysis that is capable of accomplishing both known genotype identification and novel genotype detection. Specifically, this novel surveillance functionality is achieved through time-series modeling of sequence-defined HRM curves, which is uniquely enabled by the large-scale melt curve datasets generated using our high-throughput digital HRM platform. Taking the detection of bacterial genotypes as a model application, we demonstrate that our algorithms accomplish an overall classification accuracy over 99.7% and perform novelty detection with a sensitivity of 0.96, specificity of 0.96 and Youden index of 0.92. Since HRM-based DNA profiling is an inexpensive and rapid technique, our results add support for the feasibility of its use in surveillance applications.

**Supplementary Information:**

The online version contains supplementary material available at 10.1186/s12859-024-05747-0.

## Introduction

Effective large-scale monitoring and surveillance of novel pathogens are critical components of contemporary public health strategy [1, 2]. Genotypic screening of pathogens, in particular, enables researchers and clinicians to gain nuanced insights into disease transmission patterns, virulence factors, antibiotic resistance profiles, and potential outbreak sources [3]. Modern techniques for genotypic screening, however, can be cost-prohibitive, slow, and intractable to scale [4]. High Resolution Melting (HRM) analysis offers an emerging alternative that enables rapid, effective, and economical post-amplification nucleic acid characterization for profiling DNA sequences [5–11].

HRM involves the use of a DNA-binding dye added to DNA samples, which fluoresces upon intercalating into the double-stranded structure. As the sample is heated, the DNA denatures, resulting in a loss of fluorescence which is recorded to produce a melt curve. Recent advancements have positioned HRM as not just a check on amplification product homogeneity, but also as a reliable method for heterozygote detection [5, 12]. Moreover, with improvements in heat transfer and reaction engineering, homozygous melt curves can now be leveraged as sequence-specific signatures [13–16].

When applied to the problem of identifying and differentiating bacteria, universal primers have been used to target the 16S rRNA gene, which is highly conserved in bacteria but contains variable regions that are specific to different species or strains. This enables broad-based amplification of the bacterial 16S gene with universal primers, while relying on melt to genotype the hypervariable sequences specific to organism identity. The use of machine learning (ML) classification, where each unique sequence-specific curve signature represents a pathogen class, offers a principled framework to utilize HRM as a broad-based sequence profiling tool. This could be especially valuable in clinical diagnostics, where specifically identifying pathogenic bacteria is crucial for determining the appropriate treatment [17–19].

The growth in the amount of HRM data available for ML training has further accelerated the practical application of these methods for pathogen profiling. The rising prominence of digital PCR has led to the evolution from traditional HRM to digital HRM (dHRM) which enables 200-fold increases in the number of melt curves [20, 21]. This methodology originated from our prior work in which we introduced a unique dHRM platform that employs specialized heat transfer and imaging mechanisms to simultaneously melt thousands of digital PCR (dPCR) reactions [13, 14, 22]. A distinct feature of this methodology is its digital design, in which each reaction is characterized by the presence or absence of a genome as its DNA template. While traditional HRM might typically operate on a 96-well plate, our dHRM technique utilizes a dPCR chip with 20,000 partitions, resulting in 20,000 HRM curves.

Existing literature has demonstrated effective application of Naïve Bayes (NB) [23], Support Vector Machines (SVM) [15, 16], k-Nearest Neighbors using Dynamic Time Warping [24], Random Forest (RandF) [25, 26], and Neural Networks [27], for the purpose of pathogen classification using melt curve data. SVM algorithms have shown notable performance even with relatively few melt curves. However, in the traditional one-versus-all application for multiclass classification, these methods are ill-suited to address melt curves that lie outside the distribution of their training set. Specifically, when presented with an out-of-distribution melt curve, these classifiers may erroneously classify the signature of an emerging pathogen as a known pathogen class. Thus, to enable broad-based pathogen surveillance, there is substantial promise in ML that can accurately identify the emergence of novel genotypes.

In this work, we leverage the massive datasets generated from our dHRM platform and broad-based 16S gene amplification strategy to evaluate the performance of common ML classifiers in the identification of novel and known bacterial genotypes. To our knowledge, this work represents the first study in which multiple broadly applicable ML classifiers have been investigated for the purpose of novelty detection with HRM. Previous work by Andini et al. utilized Naïve Bayes and a custom distance metric based on the Hilbert Transformation, but this approach was limited in efficacy since it aligned melt peaks to a single temperature [23]. We hypothesize that by incorporating a larger HRM dataset than previously reported, extracting significant geometric features of the melt curves, selecting the optimal ML algorithm, and developing an appropriate metric of model confidence, we can more accurately automate the identification of novel genotypes. We test this hypothesis using both experimental samples and simulated melt curves generated using the uMelt tool [28]. We focus on the specific use-case of bacteremia in neonates in which a small number of bacterial organisms are typically implicated, but emerging and opportunistic infections can occur [29].

## Material and methods

### Bacterial strains

The bacterial species used in this study and their corresponding melt curves are described in Additional file 1: Table S1. These bacteria are the primary causative pathogens in cases of neonatal sepsis [29, 30]. We obtained isolates from Dr. David Pride (University of California San Diego School of Medicine) as well as the American Tissue Culture Collection (ATCC, Old Town Manassas, VA). Bacteria were cultured in Lurie-Bertani (LB) broth or Tryptic Soy broth (TSB), as required, and incubated overnight at 37 °C.

### Bacterial genomic DNA extraction and PCR

Following culturing, we performed DNA extraction using Wizard Genome DNA Purification kit (Promega Corporation, Madison, WI). We assessed the quality and concentration of the extracted DNA using spectrophotometric absorbance measurements and confirmed the identities of the species from sequencing. We prepared genomic DNA dilutions for use with dPCR and used the commercially available QuantStudio 3D Digital PCR 20 K chip v2 (Applied Biosystems, Foster City, CA) for amplification. We followed the manufacturer’s recommended process, but customized our reagents. The composition of our dPCR master mix is described in our prior work. It includes 1 μL of sample, 0.15 μM forward primer 5′-GYGGCGNACGGGTGAGTAA-3′ (Integrated DNA Technologies, Coralville, IA), 0.15 μM reverse primer 5′-AGCTGACGACANCCATGCA-3′ (Integrated DNA Technologies, Coralville, IA), 0.02 U/μL of Phusion HotStart Polymerase (Thermo Fisher Scientific, Waltham, MA), 0.2 mM dNTPs (Invitrogen, Carlsbad, CA), 1X Phusion HF Buffer containing 1.5 mM MgCl2 (Thermo Fisher Scientific, Waltham, MA), 2.5X EvaGreen (Biotium, Fremont, CA), 2X ROX (Thermo Fisher Scientific, Waltham, MA), and ultrapure PCR water (Quality Biological Inc., Gaithersburg, MD) to bring the total volume to 14.5 μL. We loaded the chip by spreading 14.5µL of the master mix per the manufacturer’s recommendation. We then cycled the the dPCR on a flatbed thermocycler with the following cycle settings: an initial enzyme activation (98 °C, 30 s), followed by 70 cycles (95 °C, 30 s, 59 °C, 30 s, 72 °C, 60 s).

### DNA melt curve generation and preprocessing

The architecture of our U-dHRM device has been previously described [13, 14]. A copper plate hosts the microfluidic dPCR chip, separated by a thin layer of thermal grease to ensure efficient heat transfer. Temperature control is achieved through a thermoelectric module (TE Technology Inc., Traverse City, MI), PID controller (Meerstetter Engineering GmbH, Rubigen, Switzerland), Class 1/3B resistance temperature detector (RTD) (Heraeus, Hanau, Germany) embedded in the copper block, K-type thermocouple (OMEGA Engineering, Stamford, CT), and heat sink. We secure the device on-stage for optimal fluorescent imaging using a custom-made adapter. A Nikon Eclipse Ti microscope (Nikon, Tokyo, Japan) captures simultaneous fluorescent images from heat ramping the DNA-intercalating dye, EvaGreen (Ex/Em: 488 nm/561 nm) and the control dye, ROX (Ex/Em: 405 nm/488 nm). An automated image processing algorithm implemented in MATLAB is used to generate the melt curves. We perform background subtraction using the linear method described in [31]. This horizontally aligns the tails of the melt curves with the x-axis, to ensure they are most similar to the theoretically predicted uMelt curves.

### Feature engineering

The resulting preprocessed melt curves contain fluorescence loss values (−dF/dT) for 410 temperature steps in the range [51 °C, 92 °C]. We model this as a 1-dimensional time-series and apply a signature transform to each melt curve. The signature method is a non-parametric feature extractor that computes a series of integrals along a data path that fully capture its order and area [32, 33]. The signature method is optionally time-shift invariant and is sensitive to the geometric shape of the path. We apply the signature transform on a rolling window with a kernel size of 20 and a stride of 8 across the time-series with time and basepoint augmentations and a signature depth of 3 to generate a set of features for the downstream classification tasks.

### Machine learning model selection

We set out to compare five ML methods: Logistic Regression, Naïve Bayes (NB), Support Vector Machines (SVM), Neural Networks and Random Forest (RandF). To address correlation between the input signature features, we used L2 regularization for the logistic regression, SVM, and neural network models. At the end of this work we briefly discuss how calibrating the probabilities affects the results. We built and implemented all algorithms using the scikit-learn package within Python programming language [34]. All data and code are available on https://github.com/aboussina/dHRM-novelty-detection.

### Quantification of genotypic differences

To further assess the utility of our derived HRM signatures for the identification of distinct genotypes, we analyzed the ability of the aforementioned ML models to quantify the degree of genotypic differences between species. That is, beyond simply classifying a melt curve to a given species, we reformulated this as a regression problem where the target variable is a metric for genotypic difference. We calculated this metric by mapping our ten bacterial species on the SILVA phylogenetic tree and computing the patristic distance between the node of each species and the *E. coli* node [35]. We evaluated the performance of this regressor using the c-statistic as described in [36].

### Leave-one-group-out (LOGO) experiments

**Fig. 1:**
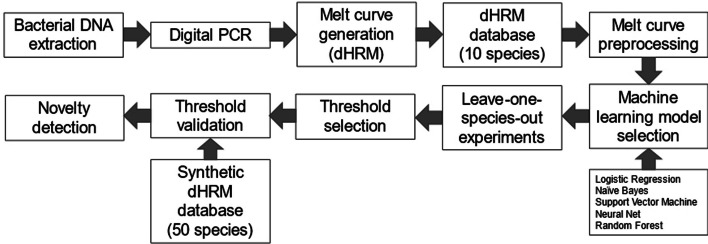
Workflow for ML novelty detection

We evaluated the capability of these machine learning models for the task of identifying novel melt curves, i.e. those belonging to species unrepresented in the training set, using a leave-one-group-out experimental design. For each of our ten bacterial species, we held out its melt curves and split the curves of the remaining nine species into training and test sets (80:20 ratio). Then, the held-out species' curves were added to the test set. The machine learning model was trained on the training set and then tested on its ability to recognize the curves of the held-out species as novel within the modified test set. This process was repeated for each of the ten species. The schematic of this approach is presented in Fig. 1. To measure our model's efficacy in novelty detection, we used Youden’s index, a metric that assesses the performance of a binary diagnostic test.

### Threshold selection

We set the threshold for novelty identification on a single LOGO experiment to the Youden Index (i.e. the point on an ROC curve at which sensitivity + specificity − 1 is maximized). However, to practically identify novel organisms, the ideal threshold should apply across all ten LOGO experiments. Thus, we calculate a ‘practical’ threshold by accumulating all the LOGO experiments and determining the Youden Index from their combined ROC curve. We then assess the performance of each LOGO experiment using this combined ‘practical’ threshold. We investigated applying sample weights to each experiment based on the number of left-out curves, but didn’t observe a significant change in the selected threshold (data not shown).

### Threshold validation with synthetic melt curves

We generated in-silico melt curves using uMelt for 50 clinically relevant bacterial pathogens, including category A and B biothreat agents and their surrogates from [37] (Additional file 1: Table S1). We added real dHRM noise to these synthetic melt curves to more realistically capture sample variation as described [26]. We created 100 melt curves per species, with a unique noise residual applied to each individual melt curve.

## Results

### Preprocessing

**Fig. 2:**
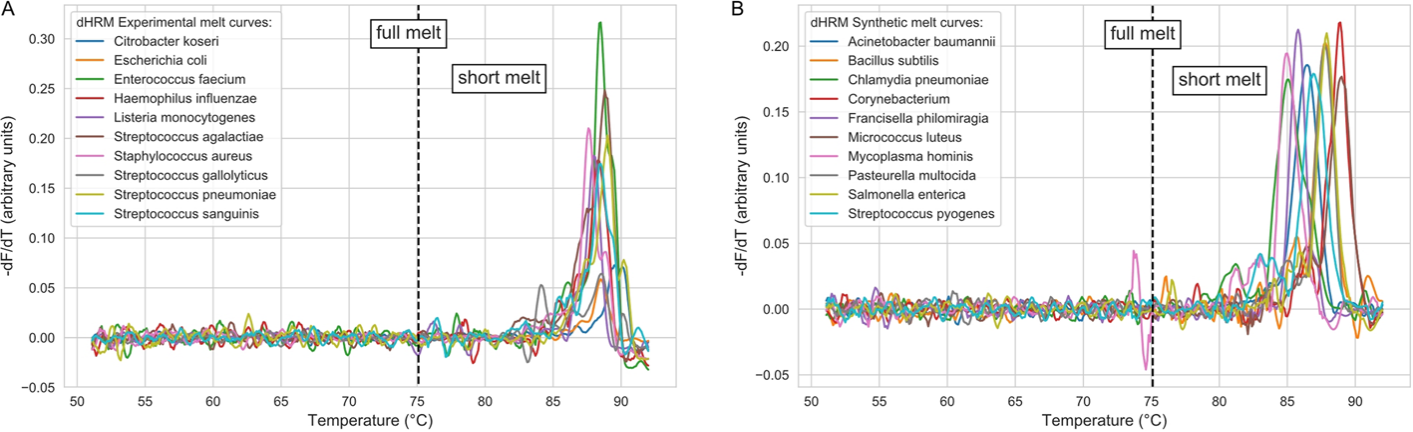
Overview of dHRM datasets. **A** Experimentally obtained dHRM melt curves. **B** Ten examples of synthesized melt curves using a combination of uMelts and real dHRM melt curve noise. Full and short melt refer to using the entire length of the melt curve or a shorter window around the melt peak location

Figure 2 and Additional file 1: Figs. S1–S2 show the results of background subtraction on the experimental melt curves. Also shown are the simulated melt curves from uMelt with added dHRM noise. Supplemental Additional file 1: Fig. S3 shows an overview of the synthetically created melt curves for all 50 pathogens. As demonstrated, the background subtraction effectively aligns the curves with the x-axis. Further, the simulated curves show similar characteristics to the experimental observations.

### Classification and regression performance

The ML methods show very similar classification accuracy, which is summarized in Table 1. No significant difference can be observed between the ‘full melt’ and ‘short melt’ classification results, which implies there might not be any additional information in the tail of the melt curve. Additional file 1: Figure S4 shows the correlation between melt curve distance (defined as the average pointwise distance from the average *E. coli* curve) and patristic distance from *E. coli*. We observe modest correlation with a couple of distinct outliers. The performance of a select ML method (RandF, n = 500) to quantify the patristic distance is listed in Additional file 1: Table S2. We observe strong performance (c-statistic: 0.96) for this regression task indicating that the derived features enable quantification of the genotypic differences between organisms.

**Table 1:** ML methods overview and classification results

	Full melt accuracy	Short melt accuracy
Logistic regression	0.996	0.997
Gaussian Naïve Bayes	0.963	0.975
SVM (rbf)	0.990	0.993
SVM (linear)	0.996	0.997
Neural net (identity)	0.994	0.997
Neural net (logistic)	0.997	0.998
Neural net (tanh)	0.996	0.998
Neural net (relu)	0.996	0.998
RandF (n = 100)	0.997	0.997
RandF (n = 500)	0.997	0.997

### Leave-one-group-out (LOGO) experiments and threshold selection

**Fig. 3:**
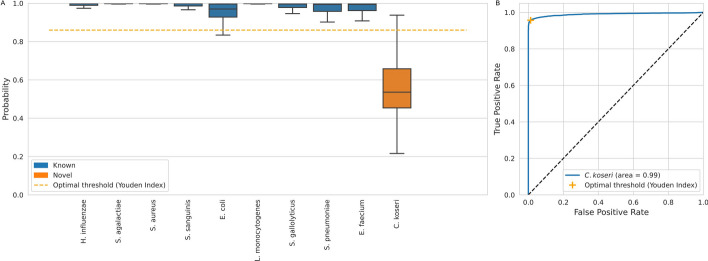
Leave-one-species-out cross validation to determine probability threshold. **A** Boxplot of the classification probabilities of each of the ten species. In this experiment *C. koseri* is the left-out species, which means it is left out of the training set and added to the test set. This experiment is repeated for each of the ten species, and an optimal threshold can be found for each of them. This experiment is repeated for all ML methods (method shown here is RandF (n = 500)). **B** ROC curve that is used to find the optimal threshold. Youden’s Index is chosen as the optimal threshold, it is the point on the ROC curve where sensitivity + specificity − 1 is maximized

Figure 3 shows the results of one LOGO experiment for one ML method (RandF, n = 500). The optimal threshold is found by plotting the ROC curve (Fig. 3B) and selecting its Youden index.

**Fig. 4:**
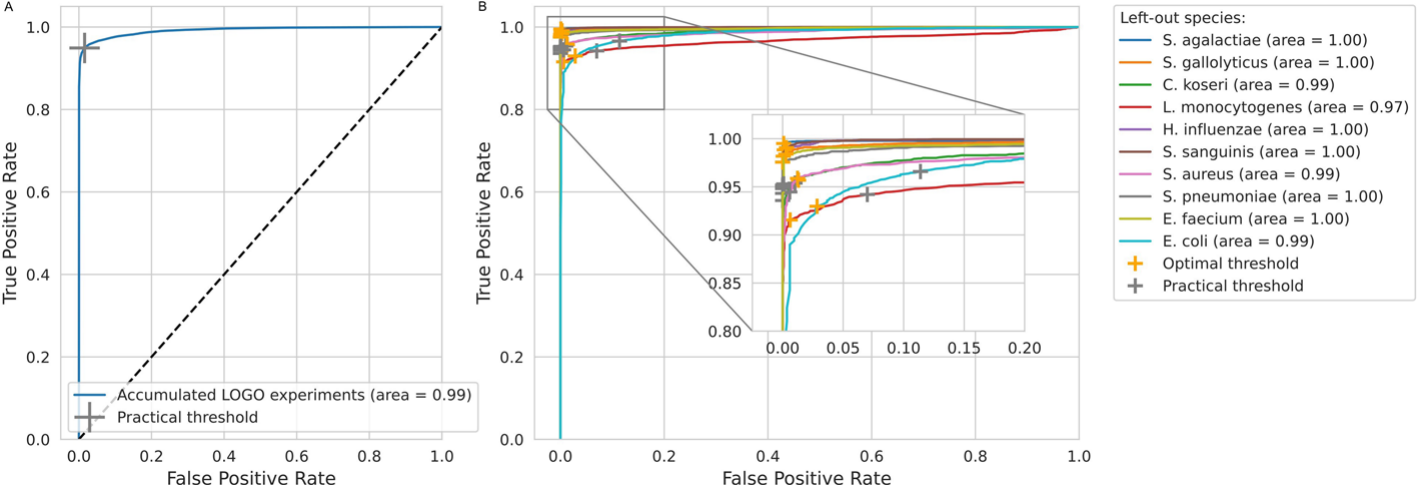
Accumulating the leave-one-group-out (LOGO) experiments results in a ‘practical’ threshold. **A** ROC curve for all ten LOGO experiments accumulated with RandF (n = 500). The optimal threshold is again found by Youden’s Index. We have named it the ‘practical’ threshold as one threshold has to be chosen (as opposed to a separate threshold for each LOGO experiment) when further validating the model on unseen ‘novel’ melt curves. It is the optimal threshold for all ten LOGO experiments combined. **B** Choosing a practical threshold implies that each LOGO experiment individually will be performing at a suboptimal threshold, which translates to a suboptimal operating point on the ROC curve

**Fig. 5:**
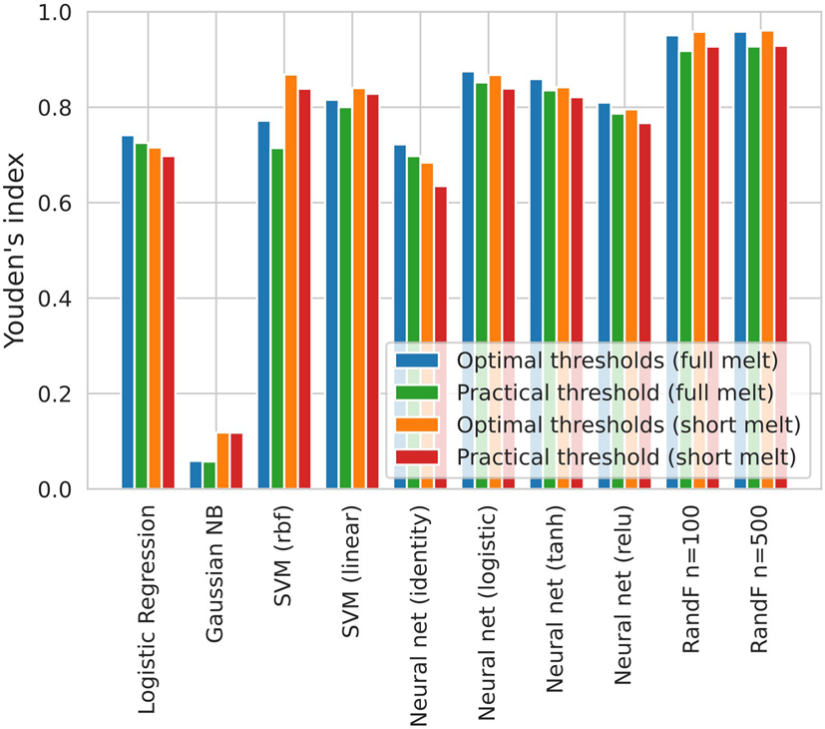
Summary of LOGO novelty detection results. Average novelty detection performance across ten species measured by Youden’s index as a function of classification method. Optimal means selecting the best threshold for each leave-one-species-out experiment. Practical means selecting one threshold and applying it to all leave-one-species-out experiments

Figures 4 and 5 summarize the results of the accumulated LOGO experiments. The ROC curves of the 10 individual LOGO experiments as well as the accumulated ROC curve are shown in Fig. 4. The performance, as measured by Youden’s index is shown as a function of classification method in Fig. 5. Each bar shows the average performance across ten LOGO experiments. ‘Optimal thresholds’ means selecting the best threshold for each LOGO experiment individually. ‘Practical threshold’ means selecting the optimal threshold for the accumulated LOGO experiments and applying it to all LOGO experiments separately. Random Forest outperforms the other methods, but Neural Nets and SVMs still perform relatively well.

### Threshold validation and novelty detection

**Fig. 6:**
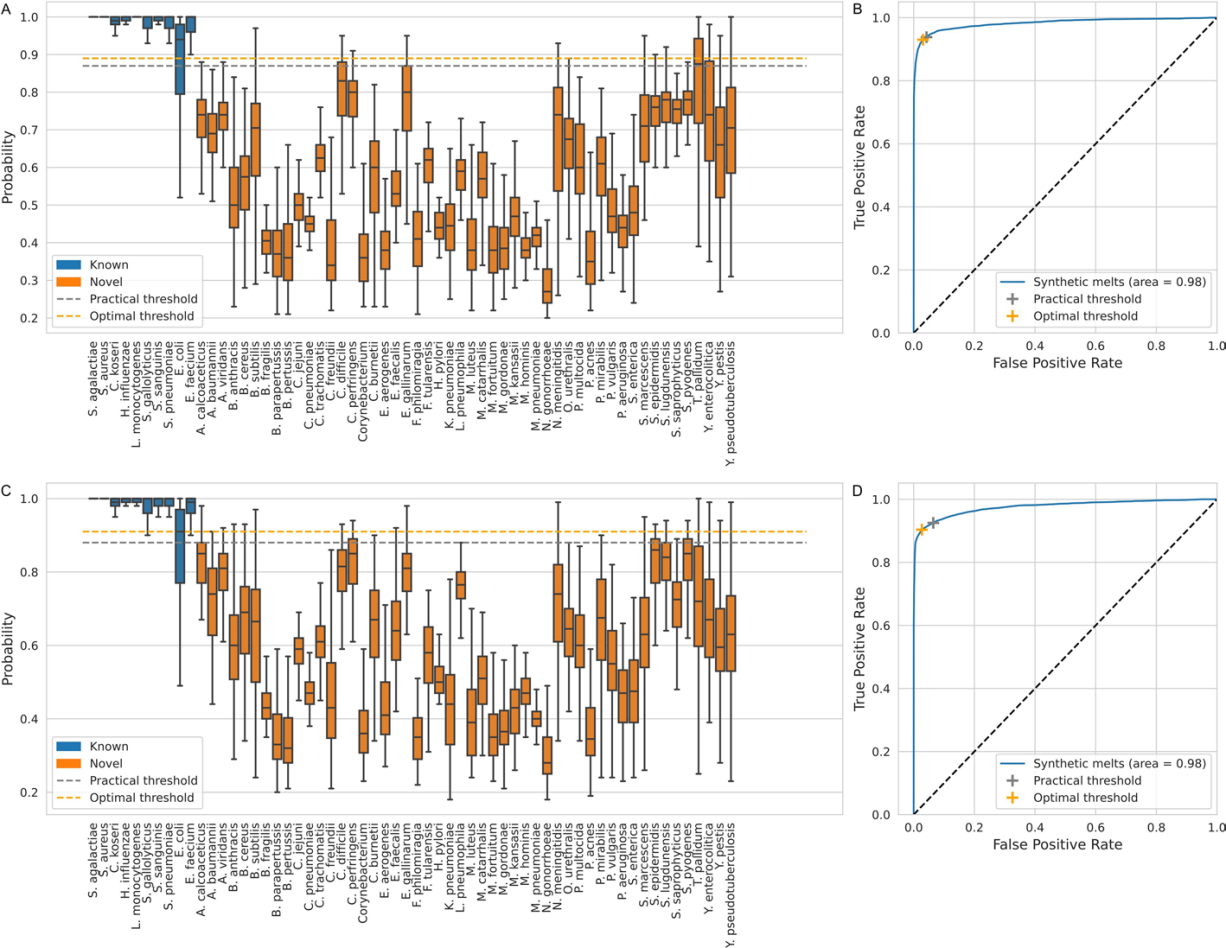
Validation of practical threshold on synthesized set of melt curves. **A** The practical threshold, selected through the LOGO experiments, was validated on the signature features from a new dataset consisting of 50 species, each with 100 synthetic melt curves with real dHRM noise. The performance of *E. coli* was confirmed to be an outlier using one-tailed t-tests. **B** ROC curve. When the practical threshold is close to the optimal threshold, it serves as a validation for the threshold selection process. **C** The practical threshold, repeated on the raw experimental and synthetic melt curves. **D** ROC curve using the raw melt curves. The ML method shown here is RandF (n = 100)

**Fig. 7:**
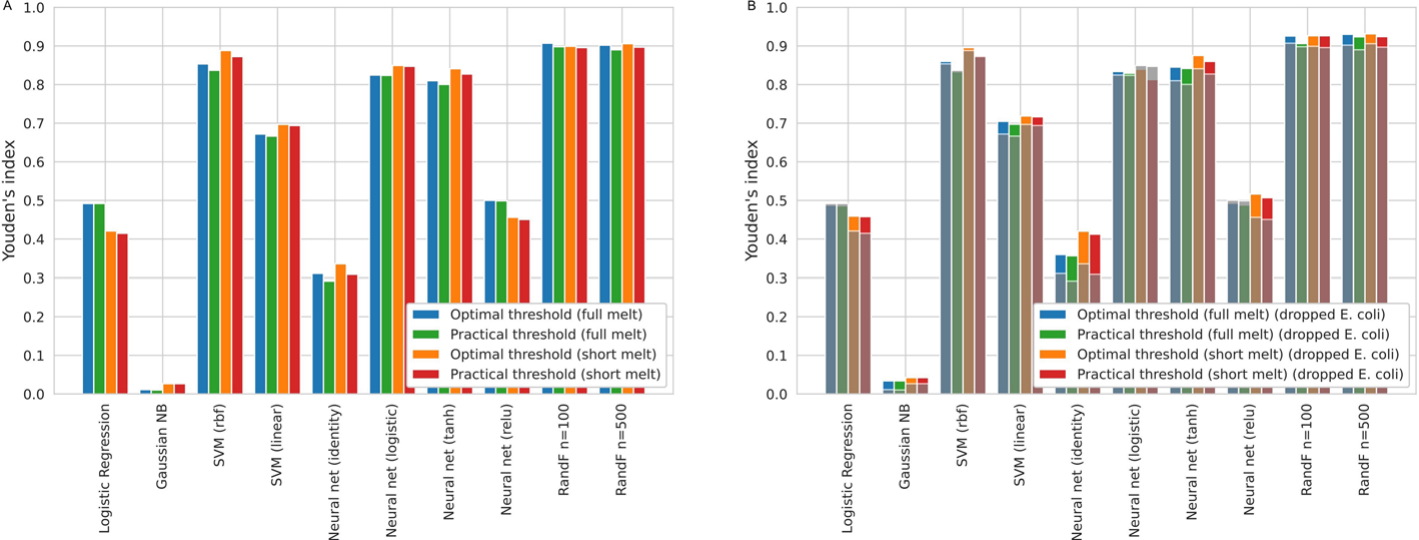
Summary of practical threshold validation results. **A** Average novelty detection performance on 50 unseen species measured by Youden’s index as a function of classification method. **B** Dropping *E. coli*, an outlier group, results in improved performance for almost all methods (results including *E. coli* are overlayed in gray). SVM (rbf) is more robust against this outlier behavior and sees less improvements

Figure 6 shows boxplots and ROC curves used to validate the previously obtained ‘practical’ threshold using the signature features (Fig. 6A, B) as well as the raw melt curves (Fig. 6C, D). The method shown in Fig. 6 is RandF (n = 100), which was one of the best performing methods, similar figures for all other methods are available in the supplementary data (Additional file 1: Fig. S5). When the practical threshold is close to the optimal threshold, the practical operating point on the ROC curve (Fig. 6B) will be close to the optimal one (Youden’s index). When this is the case, it confirms that our proposed method for obtaining a practical threshold for novelty detection, is indeed valid. Both feature sets (signatures and the raw melt curves) achieve strong discriminative performance of novel organisms based on the model score. The use of signature features enables an overall slight improvement but notably results in reduced novelty detection of T. pallidum. Figure 7A shows an overview of the results for all ML methods. The average difference between the optimal and practically attained Youden index across the ten ML methods is just 0.019 with a standard deviation of 0.025. This serves as a confirmation of our threshold selection process using the accumulated LOGO experiments. Random Forest and SVM (rbf) perform the best, with the Neural Nets a close third.

### Further improvements

We observed that the classification probabilities for the *E. coli* group of melt curves are more spread out (Fig. 6A) and might be an outlier group compared to the other species. This was apparent for multiple ML methods and was confirmed with one-tailed t-tests (e.g. for the short melt curves: P < 0.01 for Logistic Regression, Gaussian NB, SVM (rbf and linear), Neural Net (identity and relu) and RandF (n = 100 and n = 500)). As a result of this, we ran all steps again, but this time leaving out *E. coli*, to see if we could further optimize our novelty detection method. Figure 7B shows that results do indeed improve when leaving *E. coli* out. Random Forest and SVM (rbf) are the top performers, and their results are further summarized in Table 2. The best performance achieved is a Youden index of 0.93, corresponding to a specificity of 0.97 and sensitivity of 0.96.

**Table 2:** Overview of best results

	Optimal specificity	Optimal sensitivity	Optimal Youden	Practical specificity	Practical sensitivity	Practical Youden
*Full melt*						
Support vector machine (rbf)	0.93	0.93	0.86	0.96	0.88	0.83
Random forest (n = 100)	0.98	0.95	0.93	0.94	0.96	0.91
Random forest (n = 500)	0.98	0.95	0.93	0.96	0.96	0.92
*Short melt*						
Support vector machine (rbf)	0.94	0.95	0.90	0.96	0.91	0.87
Random forest (n = 100)	0.97	0.96	0.93	0.97	0.96	0.93
Random forest (n = 500)	0.98	0.95	0.93	0.96	0.96	0.92

We also investigated whether calibrating the probabilities using scikit-learn’s ‘CalibratedClassifierCV’ function would improve the outcome. We tested both the ‘sigmoid’ method, which corresponds to Platt’s method (i.e., a logistic regression model) or the ‘isotonic’ method, which is a non-parametric approach. Results are summarized in supplemental Additional file 1: Fig. S6. As expected, we see a large improvement for Naïve Bayes. We also see a significant improvement for Logistic Regression. None of the calibrated methods outperform the best results (SVM, Neural Net, RandF) as outlined in Fig. 7 and Table 2 though.

## Discussion

Our work demonstrates the utility of time-series classification algorithms in resolving multiple bacterial organism melt curves, and in identifying previously unknown (novel) melt curves that are not represented in the database. The large amount of dPCR chip-generated melt curve data enabled the development of machine learning classifiers and novelty detection algorithms, which distinguishes this study from previous studies which did not assess out-of-distribution data and utilized small datasets of melt curves [38, 39].

The only other published method specifically aimed at melt curve novelty detection [23] aligns the melt curves to one specific temperature, losing the useful melt peak location information in the process. We have selected the most widely used ML methods in HRM analysis and have shown that they are all able to classify our dHRM database with very high accuracy. Interestingly, some drastically outperform others when it comes to novelty detection. We find that Neural Nets, SVMs, and Random Forest outperform the other ML methods, even after calibrating the probabilities. Random forests utilizing features extracted from a time-series have been shown to perform well on time-series classification tasks [40]. Here, we show that its well-calibrated probabilities are also particularly useful for conducting HRM novelty detection.

The performance of our approach was improved with the removal of data for the outlier species *E. coli*. One reason for the lower performance of *E. coli* compared to the other species could be that is has the lowest number of melt curves available (Additional file 1: Table S1), which results in a smaller amount of training data available for the ML methods. We do not expect *E. coli* to inherently have more heterogeneity in its sequence compared to other species. Melt curve shape variance might be another contributor to its outlier behavior as it has the third most variance in shape (from the ten species) as measured by dynamic time warping (DTW), in our previous work [26].

No major differences were found between using the full length and short version of the melt curves, although for most methods the short version does outperform the full length, showing that there might not be any additional information in the low-temperature tail of the curve, and including it might even confound the novelty detection performance.

There are several limitations to our work. First, while we benchmarked novelty detection across a suite of machine learning algorithms, we did not perform hyperparameter tuning. It is possible that approaches such as Bayesian optimization could improve novelty detection performance. Further, we did not utilize any distance-based metric for evaluating out-of-distribution novel organisms; opting instead to leverage the output scores from discriminative classifiers to select an optimal threshold. Our approach provides an effective way to incorporate novelty detection within a large machine learning framework, but future work is required to evaluate alternative distance-aware methodologies [41].

In conclusion, advances in machine learning and ‘big data’ generation are opening up more opportunities for the advancement of HRM, which due to its speed, low cost, and simplicity was already attractive. The opportunity to use HRM as a discovery tool as well as profiling technology will further advance HRM technology towards its application in research and clinical diagnostics.

### Supplementary Information

**Additional file 1.** Supplementary Figures S1–S6 and Supplementary Tables S1–S2.

## Data Availability

The datasets used and/or analyzed during the current study are available from the corresponding author on reasonable request. Bacterial 16S gene sequences were sourced from the National Center for Biotechnology Information Reference Sequence Database located at https://www.ncbi.nlm.nih.gov/refseq/.
