# Supplementary material for: Machine learning based DNA melt curve profiling enables automated novel genotype detection
Source: BMC Bioinformatics. 2024 May 10;25:185. doi: 10.1186/s12859-024-05747-0 (PMC11088152; doi:10.1186/s12859-024-05747-0)
Supplement: Supplementary file 1 — Additional file 1. Supplementary Figures S1–S6 and Supplementary Tables S1–S2. [file 12859_2024_5747_MOESM1_ESM.docx]

**Supplementary Figures and** **Tables**


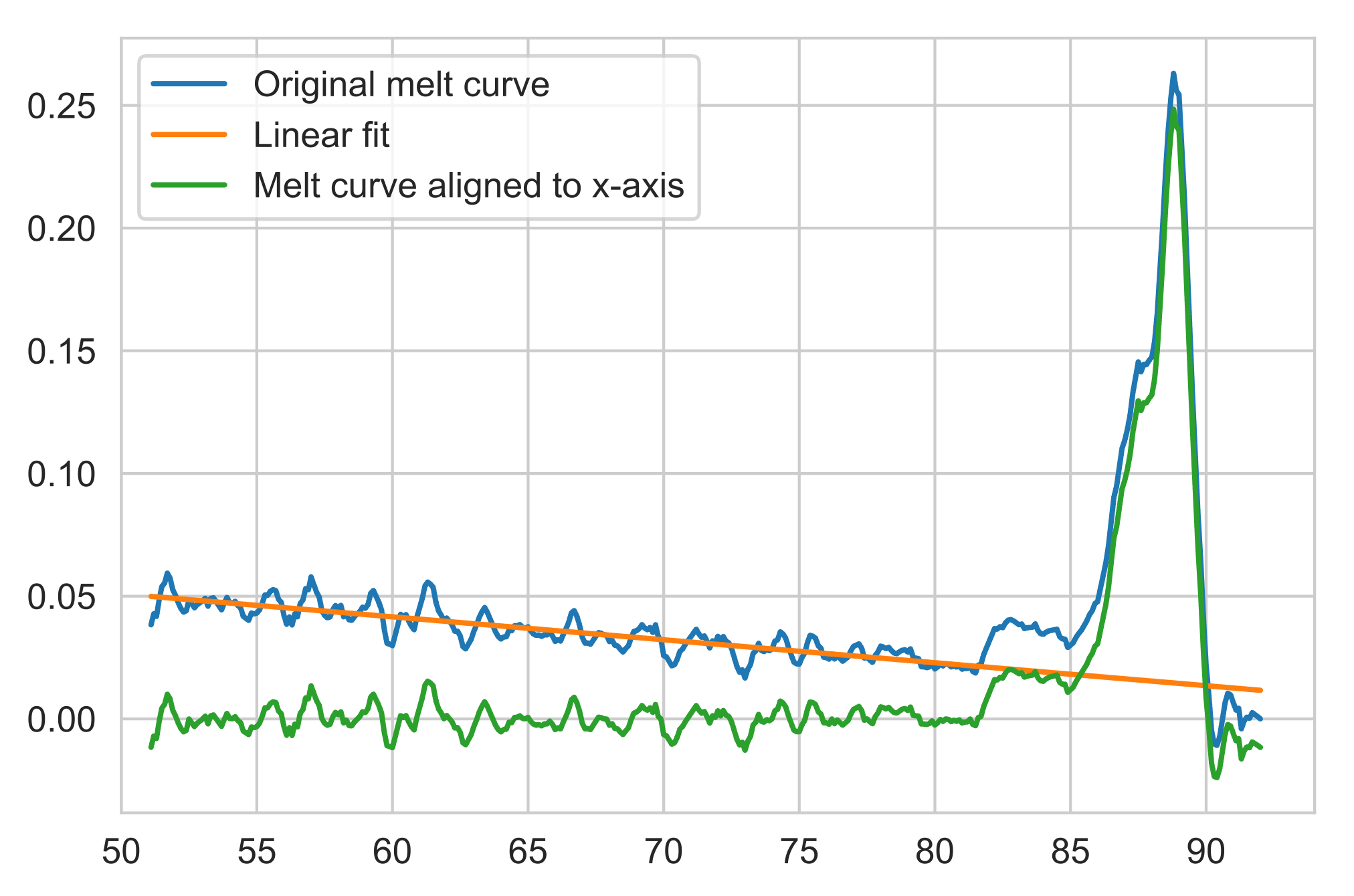

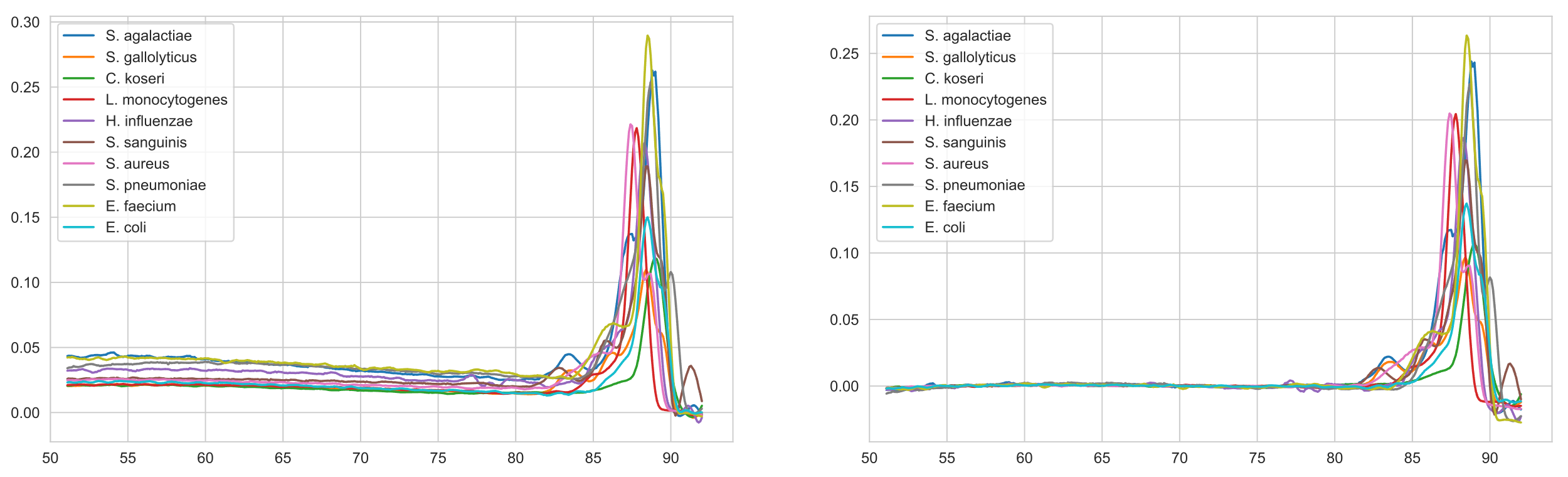


Figure S1. Data preparation. Linear background is subtracted to make sure experimental melt curves resemble synthetic ones. Top: mechanism. Left: experimental mean curves. Right: experimental mean curves after background subtraction.


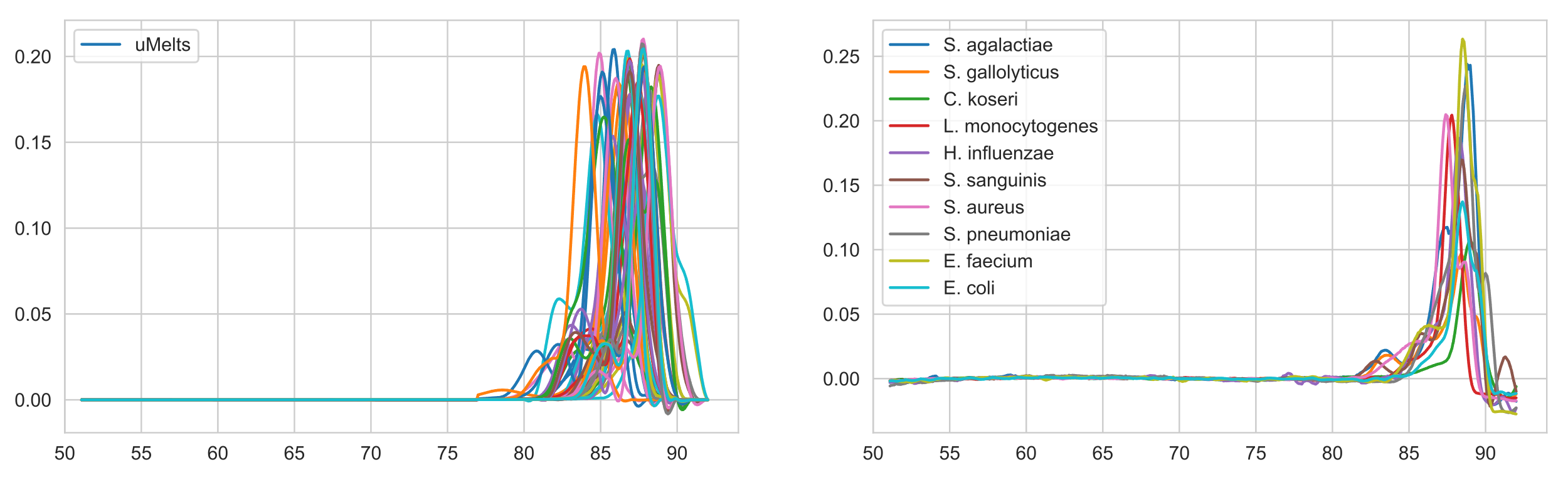

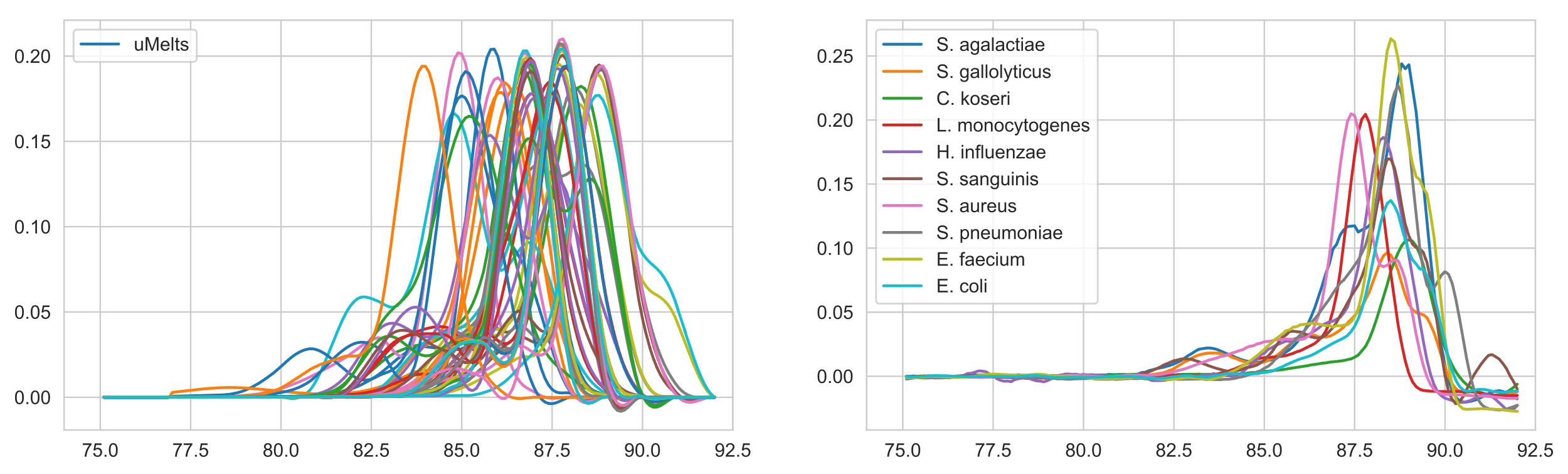


Figure S2. Overview of experimentally obtained and synthesized melt curves. Top: full melts. Bottom: short versions. For the experimental curves (right), the mean curve is shown. For the uMelts (left) the original curve is shown (without applying dHRM noise). We wanted to assure these looked similar as the noise residuals to the mean (from the experimental data) are added to the uMelts.





Figure S3. Overview of all synthesized melts. Residuals to the mean from each experimental melt curve are applied to the uMelts. The noise is scaled to the ratio of the peak heights (real peak/uMelt peak) and shifted (position real peak – position uMelt peak) so the noise should be in a similar position relative to the peak. No residual is used twice which means all the curves shown are unique. Each uMelt uses ten residuals from each of the experimental melts, which results in 100 curves per uMelt species.


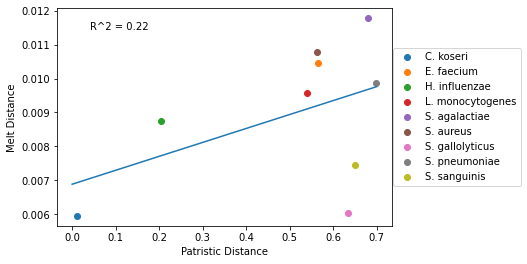


Figure S4. Correlation between melt distance and patristic distance. Patristic distance was computed for each organism by utilizing the SILVA phylogenetic tree and calculating the distance from E. Coli.


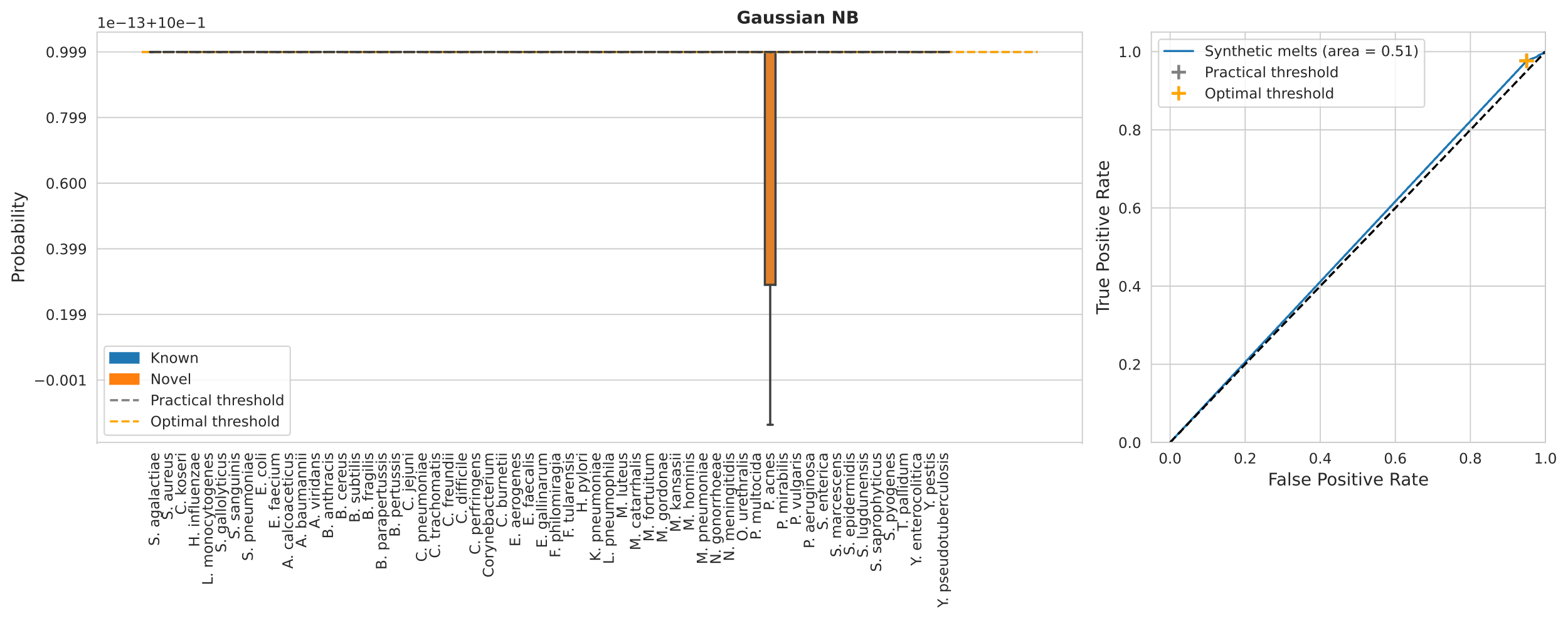

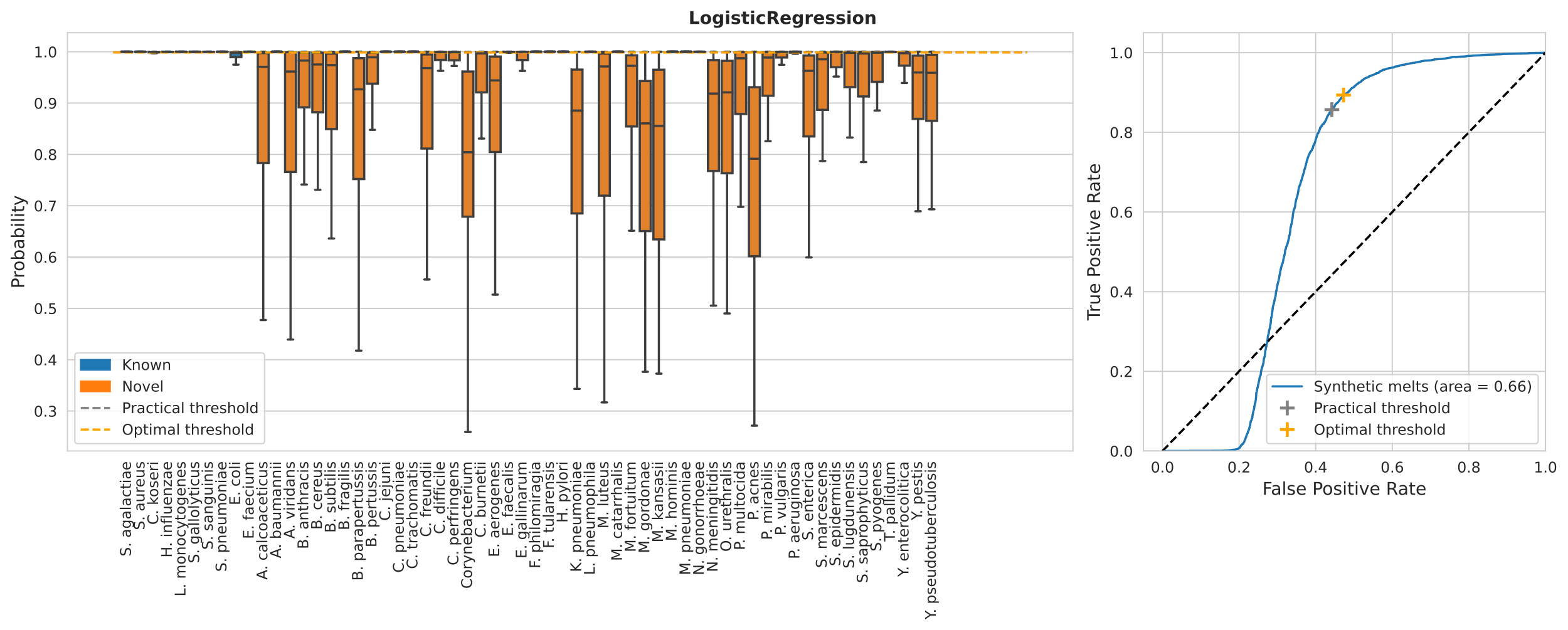


Figure S5. Validation of practical threshold on synthesized set of melt curves for all ML methods.


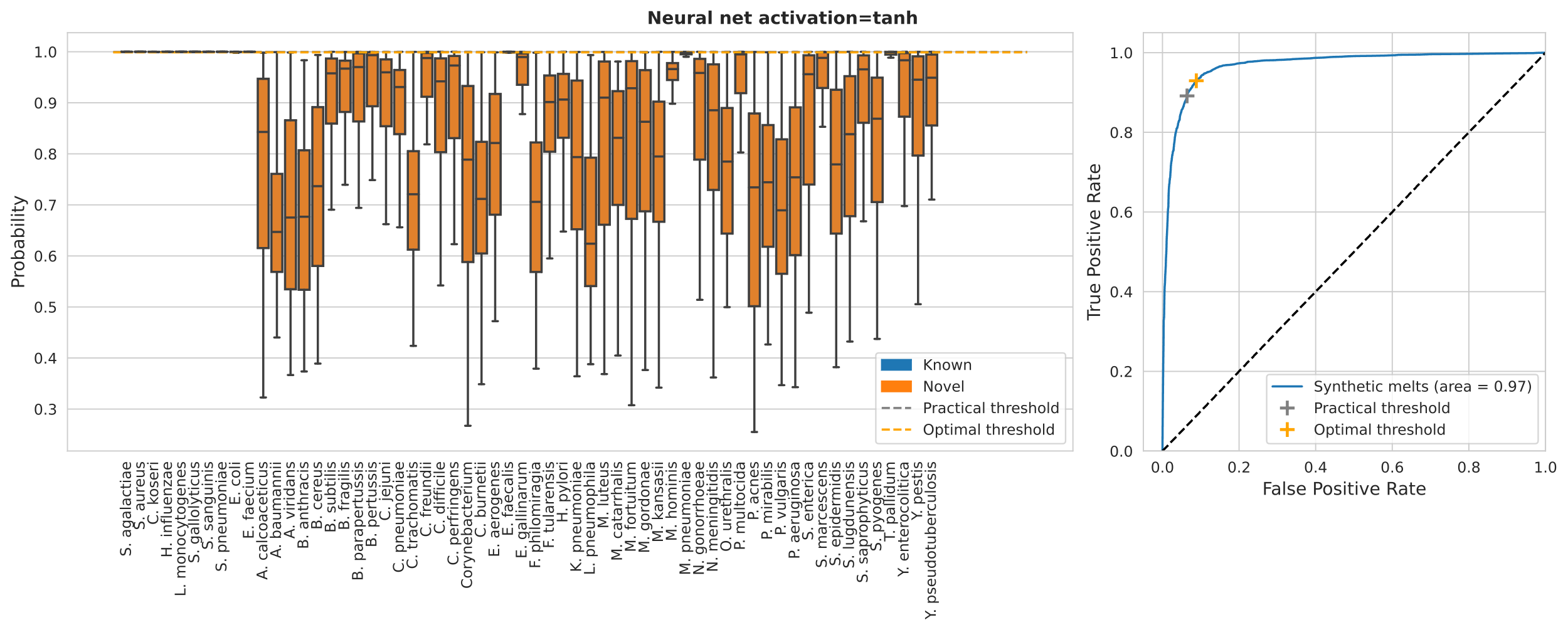

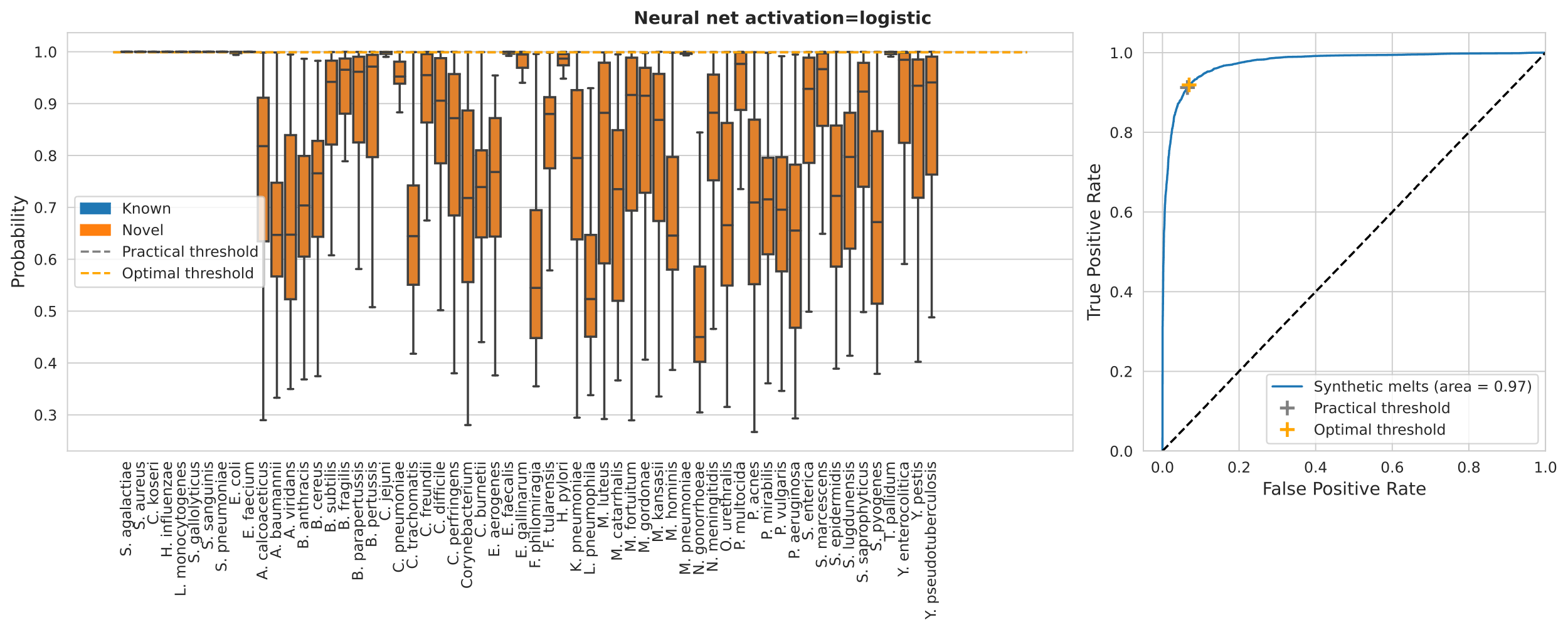

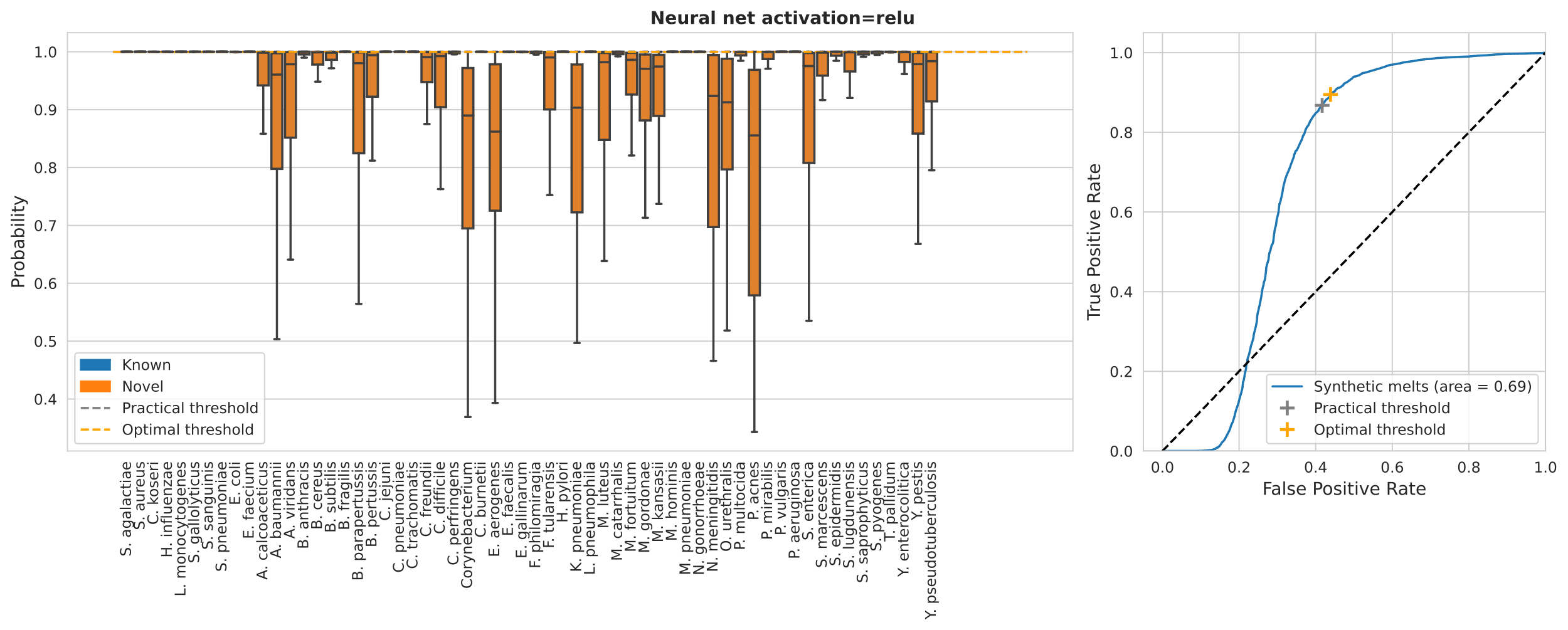


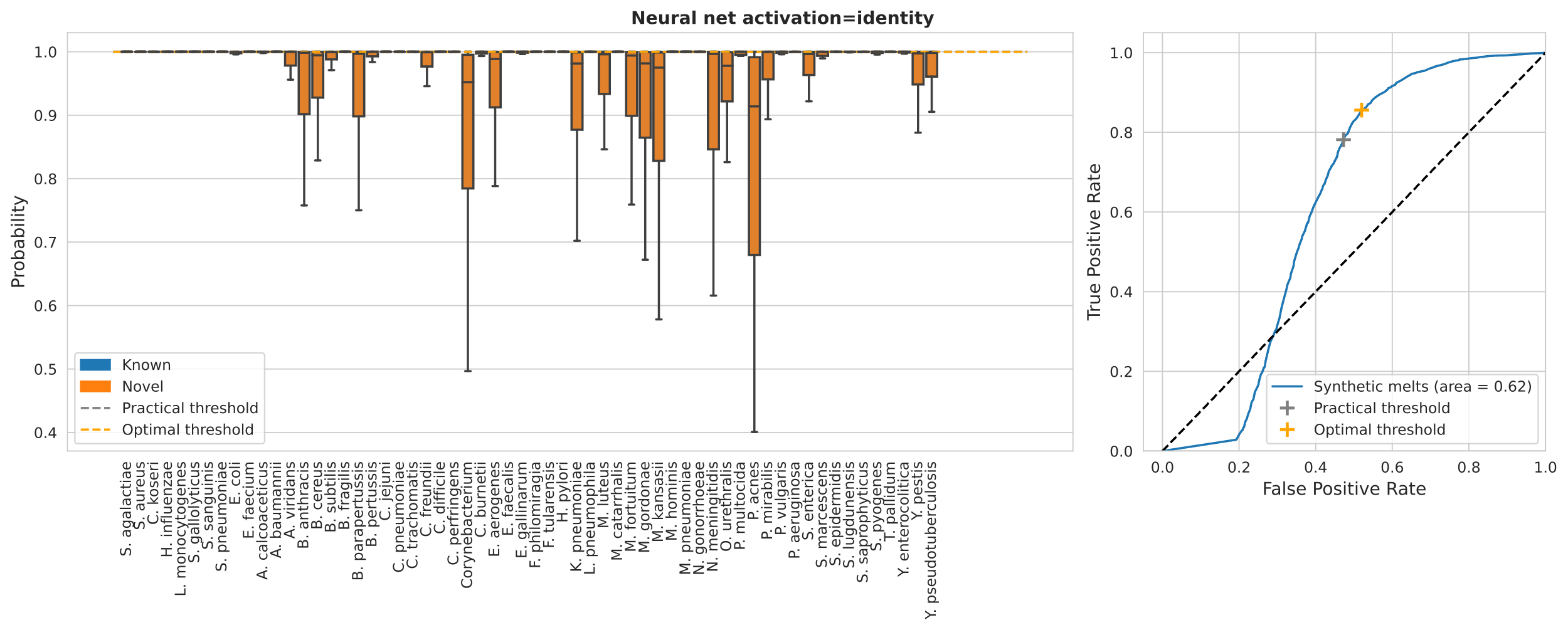


Figure S5. continued


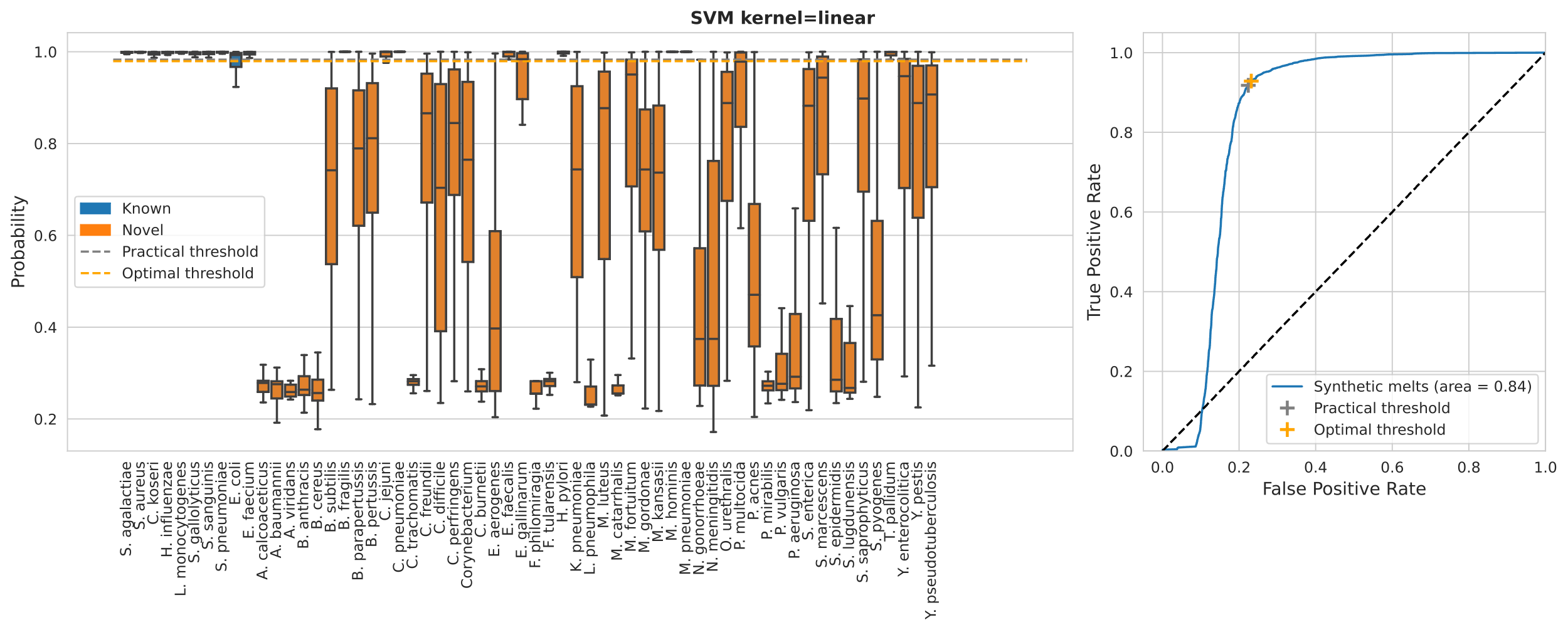

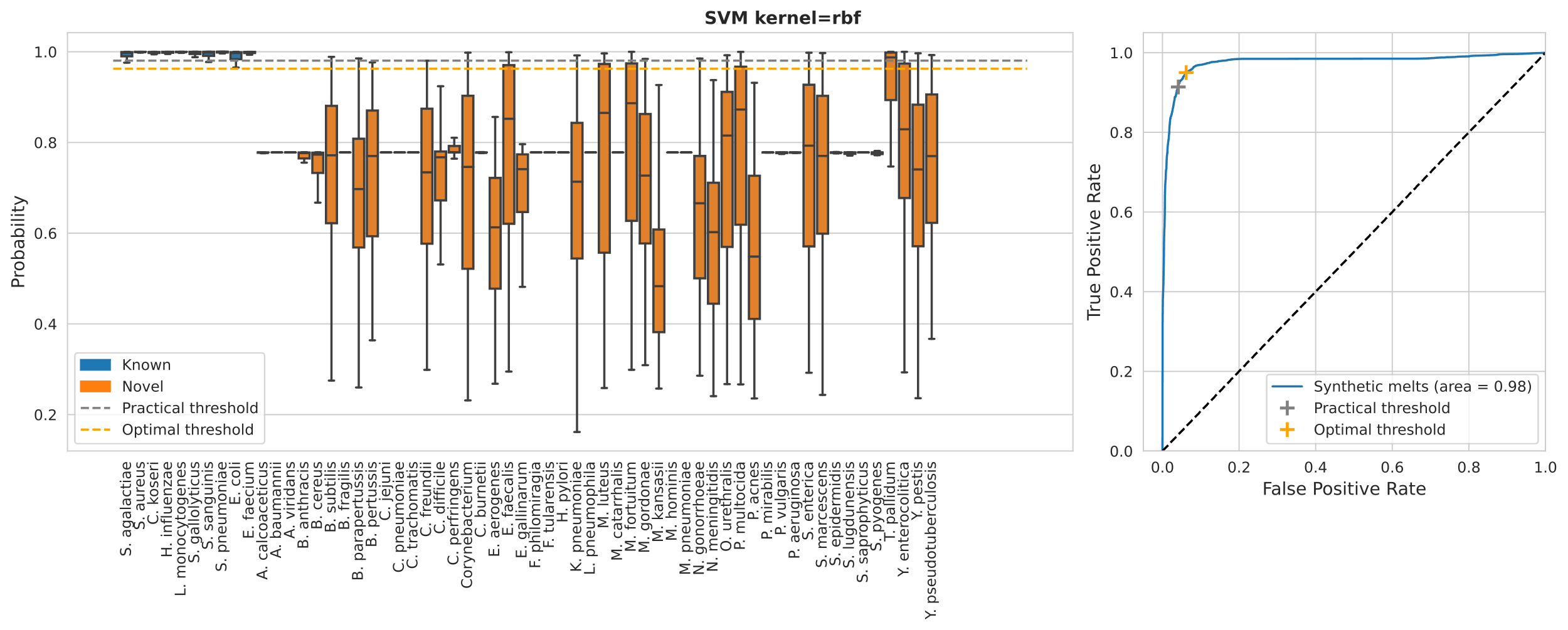

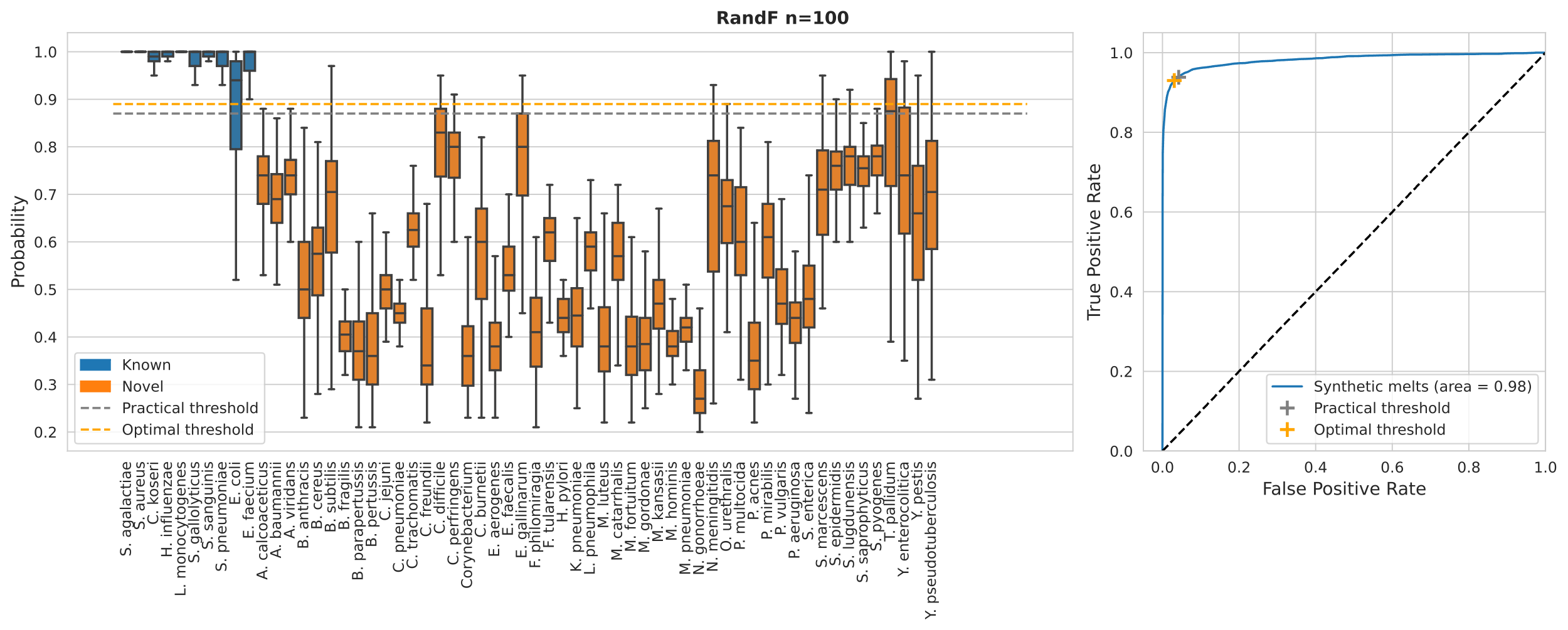


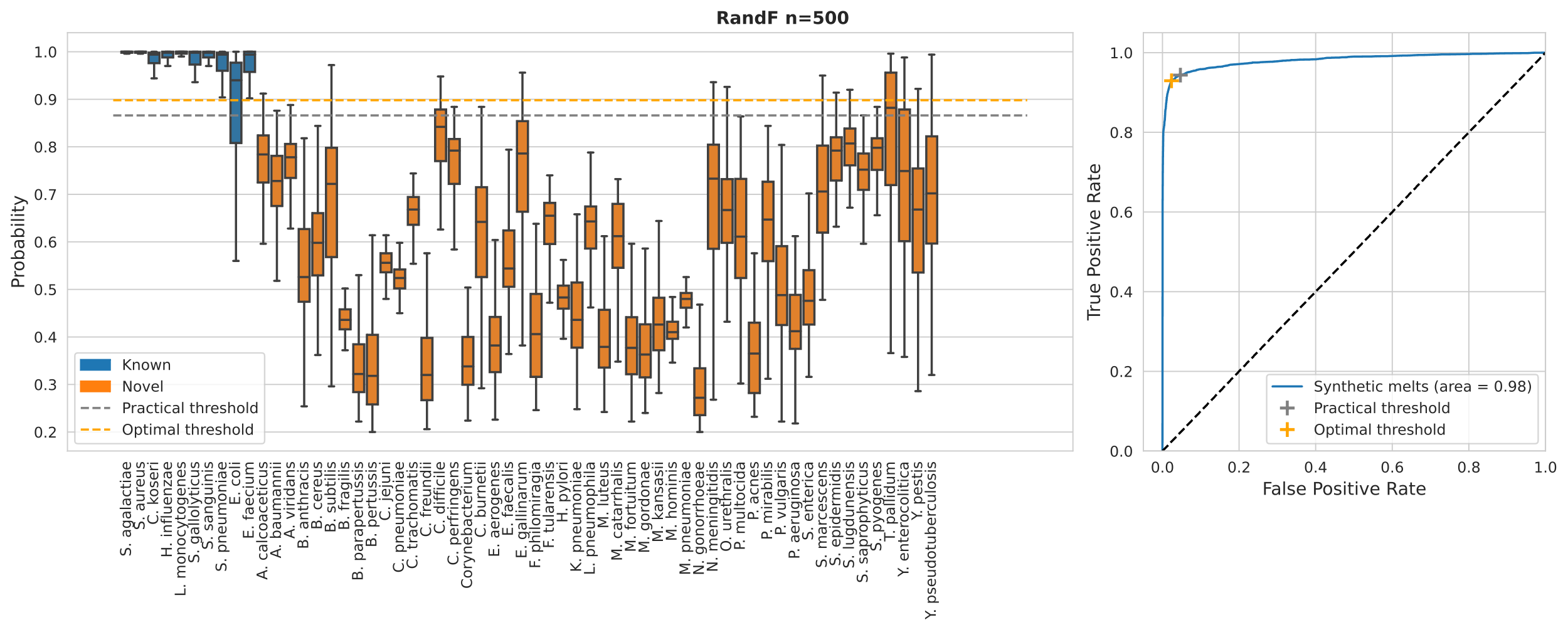
Figure S5. continued


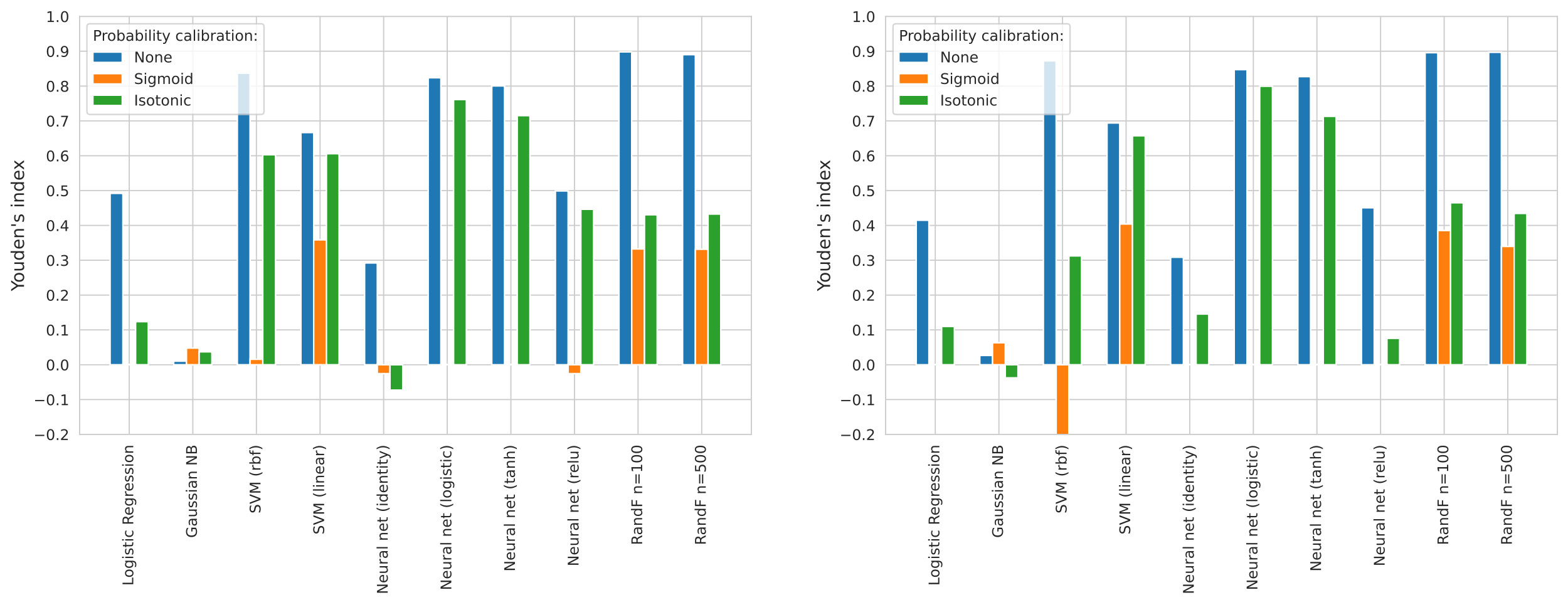

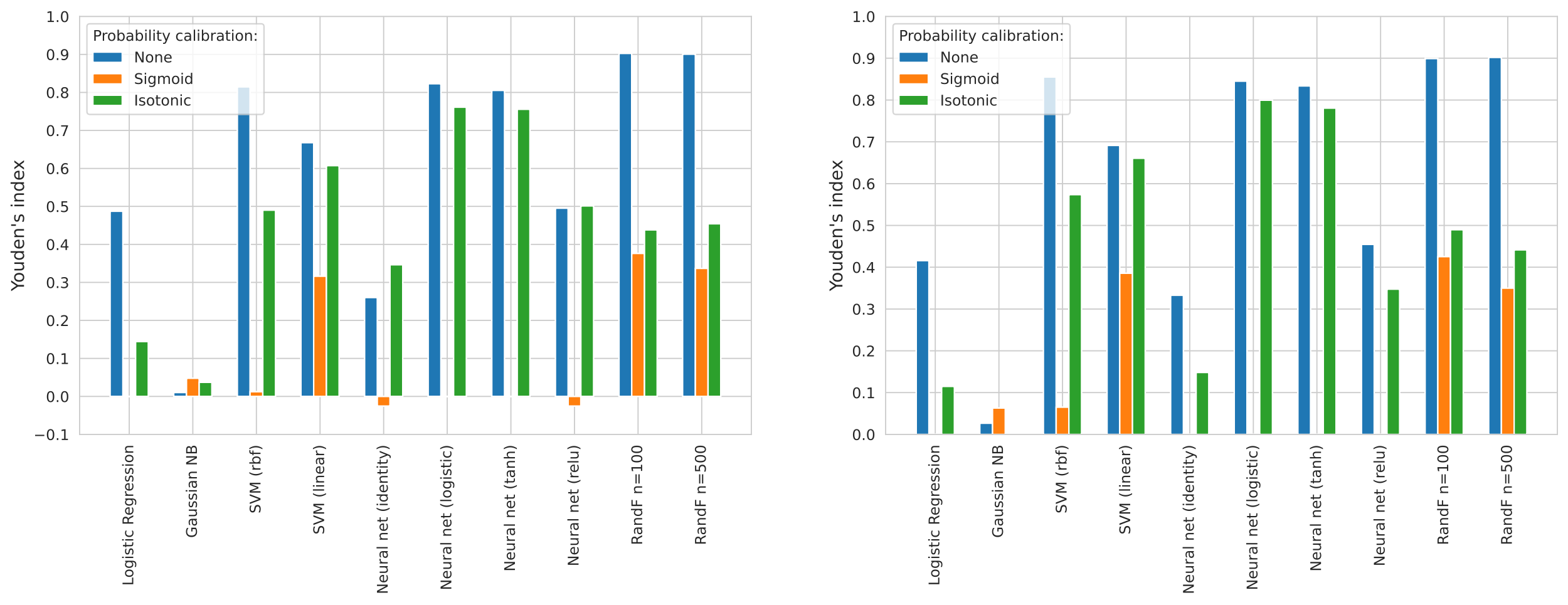


Figure S6. Probability calibration results. Top: with E. coli. Bottom: dropped E. coli. Left: full melt. Right: short melt. Calibrating probabilities improves novelty detection for Naïve Bayes. None of the calibrated methods outperform the best results (SVM, Neural Net, RandF) as outlined in Figure 7 and Table 2.

Table S1. Overview of species

| **Experimentally obtained melt curves from:**   \| Citrobacter koseri (taxid:545) \| \| --- \| \| Enterococcus faecium (taxid:1352) \| \| Escherichia coli (taxid:562) \| \| Haemophilus influenzae (taxid:727) \| \| Listeria monocytogenes (taxid:1639) \| \| Staphylococcus aureus (taxid:1280) \| \| Streptococcus agalactiae (taxid:1311) \| \| Streptococcus gallolyticus (taxid:315405) \| \| Streptococcus pneumoniae (taxid:1313) \| \| Streptococcus sanguinis (taxid:1305) \|   **Synthesized melt curves from:**   \| Acinetobacter calcoaceticus (taxid:471) \| \| --- \| \| Acinetobacter baumannii (taxid:470) \| \| Aerococcus viridans (taxid:1377) \| \| Bacillus anthracis (taxid:1392) \| \| Bacillus cereus (taxid:1396) \| \| Bacillus subtilis (taxid:1423) \| \| Bacteroides fragilis (taxid:817) \| \| Bordetella parapertussis (taxid:519) \| \| Bordetella pertussis (taxid:520) \| \| Campylobacter jejuni (taxid:197) \| \| Chlamydia pneumoniae (taxid:83558) \| \| Chlamydia trachomatis (taxid:813) \| \| Citrobacter freundii (taxid:546) \| \| Clostridium difficile (taxid:1496) \| \| Clostridium perfringens (taxid:1502) \| \| Corynebacterium (taxid:1716) \| \| Coxiella burnetii (taxid:777) \| \| Enterobacter aerogenes (taxid:548) \| \| Enterococcus faecalis (taxid:1351) \| \| Enterococcus gallinarum (taxid:1353) \| \| Francisella philomiragia (taxid:28110) \| \| Francisella tularensis (taxid:263) \| \| Helicobacter pylori (taxid:210) \| \| Klebsiella pneumoniae (taxid:573) \| \| Legionella pneumophila (taxid:446) \| \| Micrococcus luteus (taxid:1270) \| \| Moraxella catarrhalis (taxid:480) \| | **Synthesized melt curves from: (continued)**   \| Mycobacterium fortuitum (taxid:1766) \| \| --- \| \| Mycobacterium gordonae (taxid:1778) \| \| Mycobacterium kansasii (taxid:1768) \| \| \| Mycoplasma hominis (taxid:2098) \| \| \| Mycoplasma pneumoniae (taxid:2104) \| \| \| Neisseria gonorrhoeae (taxid:485) \| \| \| Neisseria meningitidis (taxid:487) \| \| \| Oligella urethralis (taxid:90245) \| \| \| Pasteurella multocida (taxid:747) \| \| \| Propionibacterium acnes (taxid:1747) \| \| \| Proteus mirabilis (taxid:584) \| \| \| Proteus vulgaris (taxid:585) \| \| \| Pseudomonas aeruginosa (taxid:287) \| \| \| Salmonella enterica (taxid:28901) \| \| \| Serratia marcescens (taxid:615) \| \| \| Staphylococcus epidermidis (taxid:1282) \| \| \| Staphylococcus lugdunensis (taxid:28035) \| \| \| Staphylococcus saprophyticus (taxid:29385) \| \| \| Streptococcus pyogenes (taxid:1314) \| \| \| Treponema pallidum (taxid:160) \| \| \| Yersinia enterocolitica (taxid:630) \| \| \| Yersinia pestis (taxid:632) \| \| \| Yersinia pseudotuberculosis (taxid:633) \| \| |
| --- | --- | --- | --- | --- | --- | --- | --- | --- | --- | --- | --- | --- | --- | --- | --- | --- | --- | --- | --- | --- | --- | --- | --- | --- | --- | --- | --- | --- | --- | --- | --- | --- | --- | --- | --- | --- | --- | --- | --- | --- | --- | --- | --- | --- | --- | --- | --- | --- | --- | --- | --- | --- | --- | --- | --- | --- | --- | --- | --- | --- | --- | --- | --- | --- | --- | --- | --- | --- | --- | --- | --- | --- | --- | --- | --- | --- | --- | --- | --- | --- | --- | --- |

**Table S2. C-statistics for patristic distance regression using random forest with 500 trees.**

|  | **Full Melt Curve** | **Short Melt Curve** |
| --- | --- | --- |
| **Raw Melt Curve** | 0.948 | 0.946 |
| **Signature Features** | 0.952 | **0.956** |
